# Efficacy and safety of sacubitril/valsartan on postoperative atrial fibrillation in adult patients undergoing cardiac surgery: a real-world observational study

**DOI:** 10.3389/fcvm.2024.1477858

**Published:** 2024-11-20

**Authors:** Xiaodong Chen, Pengxin Liu, Fengzheng Zhu, Dong Wang, Sumin Yang, Wenlong Yan

**Affiliations:** Department of Cardiac Surgery, The Affiliated Hospital of Qingdao University, Qingdao, China

**Keywords:** sacubitril/valsartan, postoperative atrial fibrillation, coronary artery bypass, valve surgery, real-world study

## Abstract

**Background:**

The mechanism underlying new-onset postoperative atrial fibrillation (POAF) in adult cardiac surgery is not well understood. However, efficient pharmacological methods to prevent and treat arrhythmic complications are still lacking. In the present study, we explored the efficacy and safety of sacubitril/valsartan (sac/val) in the control of POAF in adult cardiac surgery patients.

**Methods:**

Between January 2021 and December 2021, 667 eligible adult patients who underwent cardiac surgery at the Affiliated Hospital of Qingdao University were enrolled. The participants were divided into two groups according to whether sac/val was used: the sac/val group (*N* = 101) and the control group (*N* = 566). The main observational endpoints were the incidence of POAF, left ventricular ejection fraction (LVEF) recovery, in-hospital mortality, and short-term mortality.

**Results:**

Patients in the sac/val group had a lower incidence of POAF than those in the control group (26/101 vs. 204/566, *P* = 0.045). Patients in the sac/val group also showed a higher communicative risk for POAF incidence using the Kaplan–Meier survival analysis. In addition, patients in the sac/val group showed better LVEF recovery, with dynamic changes in LVEF superior to that of the control group. The change in LVEF in the sac/val group was 1.78 ± 5.41, compared with −1.19 ± 10.92 in the control group (*P* = 0.008).

**Conclusions:**

This is the first observational study to evaluate the efficacy and safety of sac/val in the prevention and treatment of POAF after cardiac surgery. The results demonstrated that compared with patients who did not receive sac/val treatment, those who received Sac/val treatment showed better POAF control and LVEF recovery. These results should be cautiously interpreted and further confirmed using larger sample sizes and prospective randomized controlled trials.

## Introduction

Postoperative atrial fibrillation (POAF) is the most common secondary arrhythmia after cardiac procedures (1, 2). However, POAF is not a benign disease because it increases the risk of all-cause mortality, prolongs the length of stay in the critical care unit and hospital, and increases the risk of thrombosis such as stroke (3, 4). Besides, POAF also increase the recurrence of atrial fibrillation in the years after cardiac surgery (1). However, the mechanisms underlying POAF have not been elucidated. It is unclear whether POAF is caused by a causal or a simple association. Several studies have revealed that POAF is associated with pericardial effusion and inflammation, pericardial fat metabolic activity, autonomic neurological modulation, re-entry, ectopic activity in the pulmonary vein area, ion channel modifications, gap junction uncoupling, and atrial structural alterations (5–8). Moreover, POAF also affects cardiac function, which attenuates the progression of heart failure and causes poor prognosis in patients with postoperative coronary or valve disease (9).

In clinical practice, treatment of POAF remains limited. The most frequently used antiarrhythmic medications are amiodarone and β-blockers (10, 11). However, currently, there are no specific guidelines for the treatment of POAF. The angiotensin receptor neprilysin inhibitors (ARNIs) sac/val, which perform well in clinical trials of PARADIGM-HF, are effective and safe medications for treating heart failure (12, 13). Recently, several studies have revealed that ARNI drugs not only affect ventricular remodeling but also potentially act on the control of arrhythmia (14–16). Clinical research has also shown that ARNI drugs can decrease the incidence of atrial arrhythmia or ventricular arrhythmia in patients with heart failure. The combined mechanisms for modulation of the renin-angiotensin-aldosterone system (RAAS) and sympathetic nervous system (SNS) may explain the combined effect of this drug. Besides, other medications, such as SGLT2i, were shown to have effect on the control of POAF based on meta-analysis (17). The meta-analysis revealed that Dapagliflozin use was associated with significant reduction in AF risk as compared to placebo in overall population and patients with diabetes, whereas the use of other gliflozins did not significantly reduce AF occurrence. However, there is still no study on evaluation of sac/val for controlling POAF in real world setting. Therefore, in the present study, we assumed that sac/val could prevent POAF and ventricular arrhythmia after cardiac surgery, as POAF has mechanisms similar to atrial fibrillation. Herein, we conducted this retrospectively observational study based on our single-centered real-world data, to compare the clinical effect of sac/val with control group for the prevention and treatment of POAF after cardiac surgery.

## Methods

### Study design and patient enrollment

This retrospective observational cohort study enrolled patients with cardiac diseases and heart failure at the Affiliated Hospital of Qingdao University. Patients were divided into sac/val and control groups according to whether sac/val was administered for more than 7 days. The medicine used was sac/val tablets (Novartis International AG), with a dosage range of 25–200 mg. This study was approved by the Ethics Committee of the Affiliated Hospital of Qingdao University (grant No. QYFY WZLL 28659).

### Inclusion and exclusion criteria

Inclusion criteria: (a) patients who underwent cardiac surgery at the Affiliated Hospital of Qingdao University. (b) Patients with structural cardiac malformations and diastolic cardiac dysfunction presenting with symptomatic heart failure. (c) Patients aged >18 years.

Exclusion criteria: (a) patients with a history of arrhythmia, including atrial fibrillation or ventricular tachycardia. (b) Emergency cardiac surgery. (c) Repeated cardiac surgeries (d) Congenital cardiac disease. (e) Apparent liver and/or kidney dysfunction. (f) Concomitant disease requiring long-term radiotherapy, chemotherapy, or hormone therapy. (g) Poorly controlled hyperthyroidism. (h) Hypertrophic cardiomyopathy. (i) Prosthetic ascending aortic graft implantation. (j) Concomitant cardiac tumor.

### Data collection

The observational population included patients who underwent coronary artery bypass grafting (CABG) and/or heart valve replacement or repair surgery. Baseline data, including height, weight, age, sex, blood pressure, heart rate, previous medical history, NYHA class, preoperative medication, preoperative echo, surgical procedure, incidence of POAF and/or ventricular arrhythmia, occurrence time of POAF and/or ventricular arrhythmia, postoperative amiodarone use, and major adverse cardiovascular events (MACE), were systematically collected.

### Definitions

POAF was defined as newly developed postoperative AF in patients without preoperative arrhythmia. MACE were defined as perioperative cardiac death, myocardial infarction (MI), or stroke. Patients routinely underwent ECG within 7 days after the operation. If cardiac arrhythmia occurred, an additional ECG monitoring was performed.

### Statistical methods

SPSS software (version 26.0) was used for statistical analysis. Numerical variables are presented as mean ± standard deviation, while categorical variables are presented as frequency (percentage). The *t*-test, chi-squared test, and Fisher's exact test were used to calculate differences between the sacubitril/valsartan and control groups. Kaplan–Meier survival analysis was used to calculate the incidence of time-dependent POAF, and log-rank test was used for intergroup comparisons. *P* < 0.05 (bilateral) is defined as statistically significant.

## Results

### Patients’ characteristics

**Figure 1:**
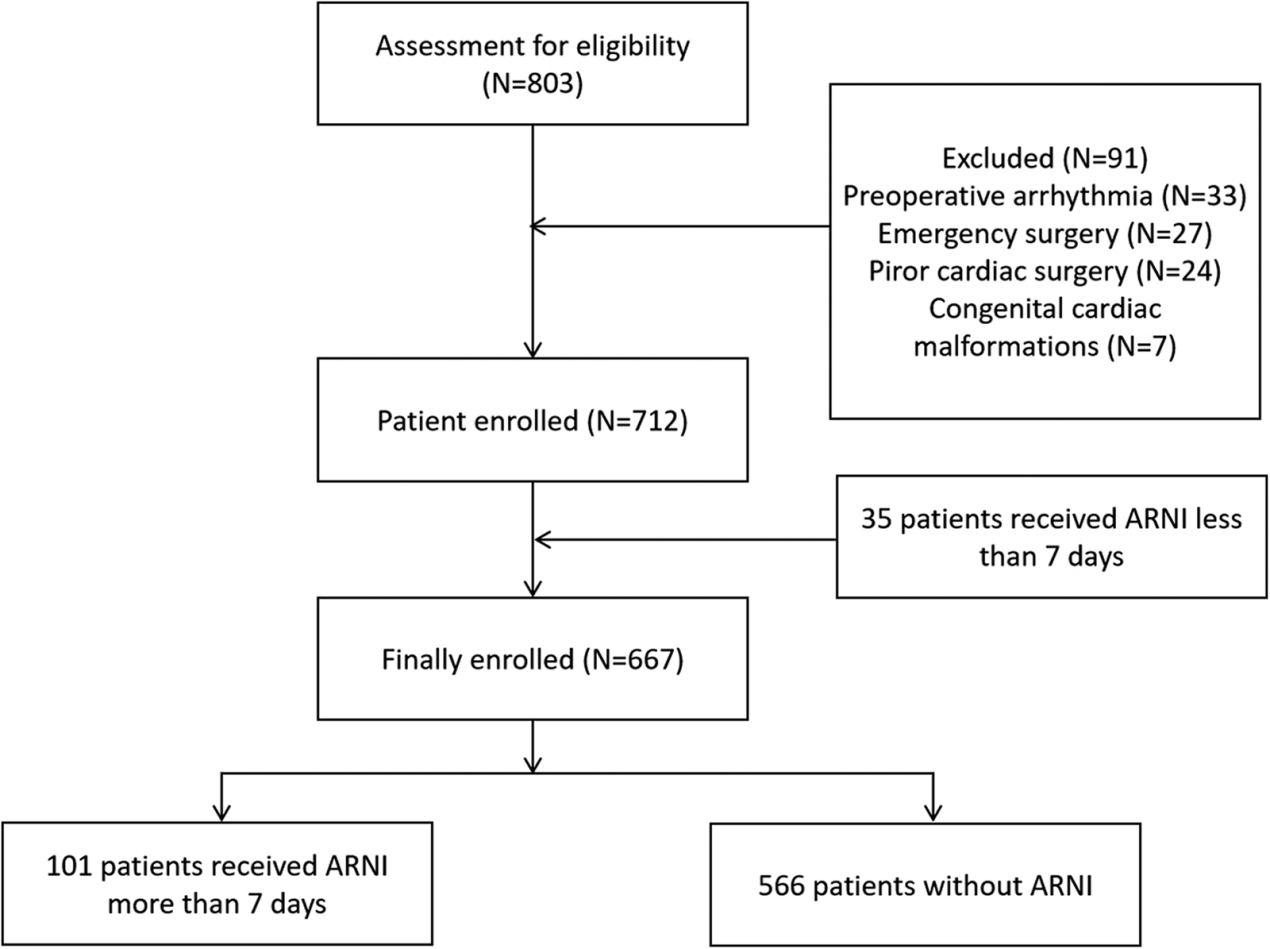
Patients’ enrollment diagram.

Between January 2021 and December 2021, 667 eligible adult patients who underwent cardiac surgery at the Affiliated Hospital of Qingdao University were enrolled. The participants were divided into two groups according to whether sac/val was used: the sac/val group (*N* = 101) and the control group (*N* = 566). A patient enrollment diagram is shown in Figure 1. The patient characteristics are listed in Table 1. Except for preoperative EF (50.74 ± 9.87 in sac/val group vs. 58.56 ± 6.66 in control group, with *P*-value <0.05), other baseline and perioperative data was comparable between sac/val group and the control group (all *P* > 0.05).

**Table 1:** Patients’ baseline characteristics.

Variables	ARNI group (*N* = 101)	Control group (*N* = 566)	*P-*value
Gender (M/F)	71/30	402/164	0.882
Age (years)	64.02 ± 8.68	63.36 ± 9.06	0.498
BSA (m^2^)^a^	1.78 ± 0.17	1.76 ± 0.17	0.272
Preoperative EF (%)	50.74 ± 9.87	58.56 ± 6.66	<0.001
Preoperative LVEDD (cm)	5.20 ± 0.59	4.91 ± 2.25	0.206
Smoking history (%)	41/101 (40.59)	176/566 (31.10)	0.061
Serum creatinine	77.19 ± 11.97	76.28 ± 13.12	0.563
Potassium	3.78 ± 0.97	3.66 ± 1.11	0.671
Previous cerebral infraction history (%)	15/101	55/566	0.121
NYHA class			
NYHA I	18	76	0.176
NYHA II	40	264	
NYHA III	36	193	
NYHA IV	7	17	

^a^ BSA (m^2^) = 0.0061 × height (cm) + 0.0128 × weight (kg) − 0.1529.

### Observational outcomes

**Figure 2:**
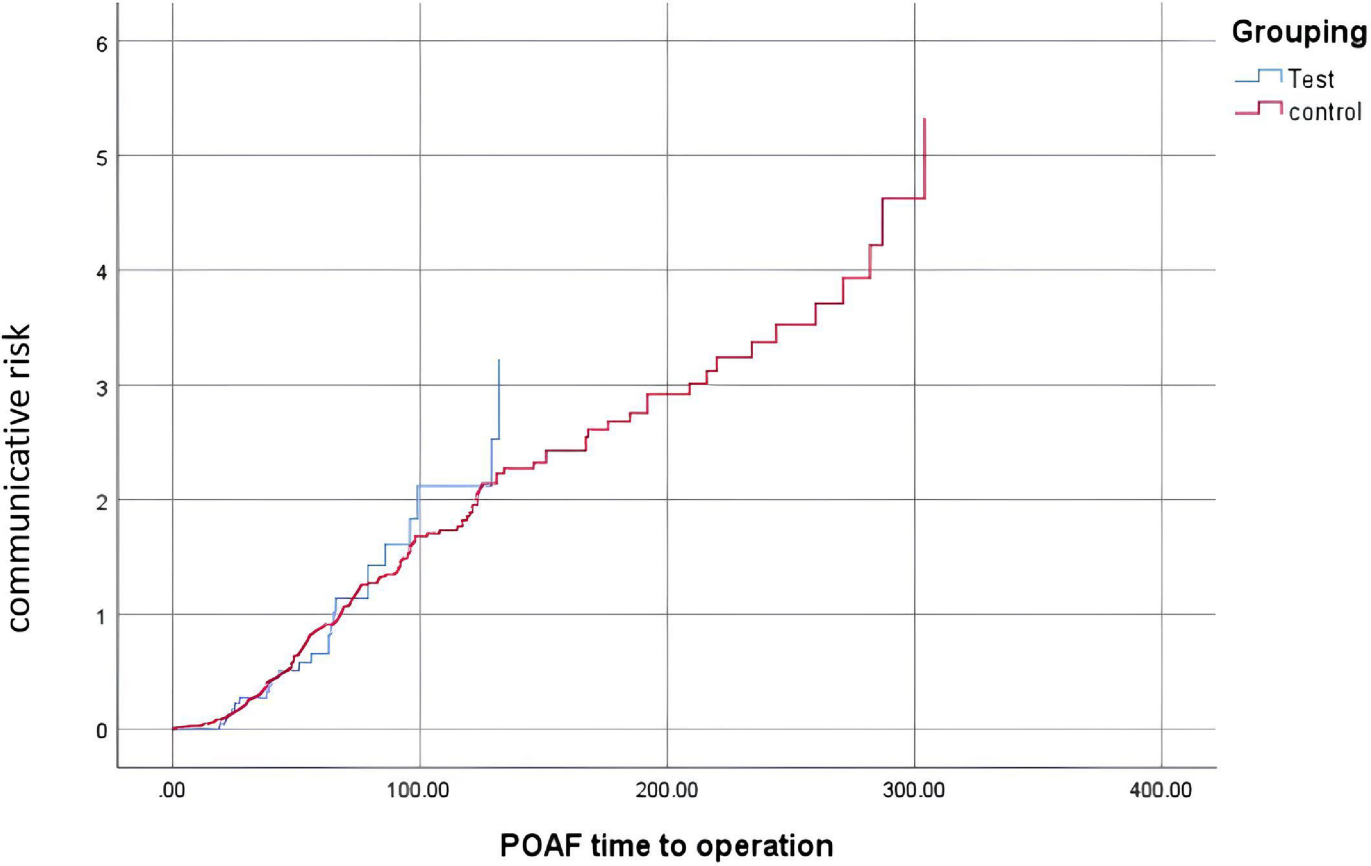
Survival analysis.

As shown in Table 2, the patients in the sac/val group had a lower incidence of POAF than those in the control group (26/101 vs. 204/566, *P* = 0.045). In addition, as demonstrated in Table 2, patients in the sac/val group showed better left ventricle function recovery, with the dynamic change in LVEF being superior to that of the control group. The change in LVEF in the sac/val group was 1.78 ± 5.41, compared with −1.19 ± 10.92 in the control group (*P* = 0.008). As demonstrated in Figure 2, patients in the sac/val group showed a similar communicative risk for the concurrence time of POAF using Kaplan–Meier survival analysis compared with the control group.

**Table 2:** Comparison of patients’ clinical outcomes.

Variables	ARNI group (*N* = 101)	Control group (*N* = 566)	*P*-value
POAF incidences	26/101	204/566	0.045
Time for POAF	64.60 ± 41.00	71.29 ± 59.72	0.587
Change in LVEF	1.78 ± 5.41	−1.19 ± 10.92	0.008
Change in LVEDD	−0.44 ± 0.48	−0.42 ± 2.33	0.935
MI incidence	2/101	12/566	0.928
CI incidence	0/101	8/566	0.229
MACE	3/101	31/566	0.291
Re-hospitalization for HF	2/101	5/566	0.781

MI, myocardial infarction; CI, cerebral infraction; MACE, major adverse cardiovascular events; HF, heart failure.

## Discussion

To the best of our knowledge, this is the first observational study to evaluate the efficacy and safety of sacubitril/valsartan for the prevention and treatment of POAF after cardiac surgery in adults. The results demonstrated that compared with patients who did not receive sac/valsartan treatment, those who received sacubitril/valsartan treatment showed better POAF control and LVEF recovery.

POAF is the most common arrhythmia after cardiac surgery and is associated with high incidence and poor prognosis (5–9). Preoperative fragility of the atrial base further increased the incidence of POAF (18). However, in clinical practice, surgeons ignore the impact of preoperative medication on the atrial base. Therefore, it is vitally important to optimize the medication strategy of patients with heart disease combined with heart failure to decrease postoperative arrhythmia complications, decrease the length of hospital stay and in-hospital MACE, and improve patients’ survival outcomes.

ARNI drugs act on POAF via several pathways. First, POAF strongly correlated with heart failure. Therefore, POAF triggers rapid and irregular movement of the heart muscles, subsequently alleviating the symptoms of patients with heart failure and promoting the progression of ventricular remodeling. In contrast, severe heart remodeling will further change the atrial base, subsequently affecting electronic signal transformation and improving the alleviation of atrial fibrillation. Therefore, ARNI drugs could both improve the ventricle remodeling, as well as have effect on different kinds of atrial fibrillation (19, 20). Second, the activation of the perioperative sympathetic nervous system (SNS) is involved in the occurrence of POAF. Therefore, ARNI drugs can inhibit enkephalinase to increase the concentration of blood natriuretic peptides (NPs). Therefore, ARNI drugs are involved in the activation of SNS (21). Third, postoperative pericardial effusion and inflammation also affect the occurrence of POAF; a more aggressive intervention indicates more severe inflammation. In several studies, the timing of POAF often overlaps with the peak C-reactive protein (CRP) level, which reflects the role of inflammation in the occurrence and progression of POAF (22). Although this study did not reveal a difference in CRP between the sac/val and control groups because most patients failed to receive regular CRP detection in real-world settings, and a false-positive peak value of CRP was detected in this study.

Overall, in this study, we found that the preoperative sac/val group was associated with a greater decrease in POAF incidence and increased LVEF recovery than the control group. Further pilot experimental studies with larger sample sizes, longer durations, and prospective randomized controlled studies should be conducted to observe the impact of ARNI on POAF in clinical practice.

## Conclusions

This is the first observational study to evaluate the efficacy and safety of sacubitril/valsartan for the prevention and treatment of POAF after cardiac surgery in adults. The results demonstrated that, compared with patients who did not receive sacubitril/valsartan treatment, those who received sacubitril/valsartan treatment showed better POAF control and LVEF recovery. These results should be confirmed by a larger sample size and prospective randomized controlled trials.

## Data Availability

The raw data supporting the conclusions of this article will be made available by the authors, without undue reservation.
